# Charge density analysis for crystal engineering

**DOI:** 10.1186/s13065-014-0068-x

**Published:** 2014-12-16

**Authors:** Anna Krawczuk, Piero Macchi

**Affiliations:** 1 Faculty of Chemistry, Jagiellonian University, Ingardena 3, Krakow, 30-060 Poland; 2 Department of Chemistry and Biochemistry, University of Bern, Freiestrasse 3, Bern, 3012 Switzerland

**Keywords:** Charge density analysis, Crystal engineering, Supramolecular chemistry, X-ray diffraction

## Abstract

This review reports on the application of charge density analysis in the field of crystal engineering, which is one of the most growing and productive areas of the entire field of crystallography.

While methods to calculate or measure electron density are not discussed in detail, the derived quantities and tools, useful for crystal engineering analyses, are presented and their applications in the recent literature are illustrated. Potential developments and future perspectives are also highlighted and critically discussed.

**Electronic supplementary material:**

The online version of this article (doi:10.1186/s13065-014-0068-x) contains supplementary material, which is available to authorized users.

**Figure:**
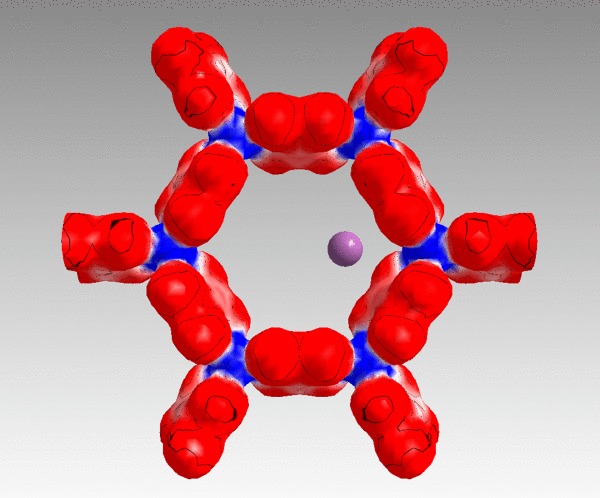
Graphical abstract

## Introduction

In modern crystallography, a crucial issue is the understanding of interactions that enable the assembly of molecules and the fabrication of flexible or rigid organic or metal-organic polymers.

The supra-molecular paradigm is often associated with *crystal engineering*. This name was originally introduced by Pepinsky [1], but later used by Schmidt [2] with a different meaning namely the usage of crystals for controlled stereospecific chemical reactions. In Schmidt’s view, the crystal is a matrix in which the reaction occurs and, at the same time, a precursor of the desired material. On the other hand, crystal engineering has later evolved towards the rationalization of binding motifs and their usage to create crystalline materials with specific structural or functional features [3]: the crystal and its structure have become the subject themselves of the speculation and the target of the research. Crystal engineering is the initial and fundamental step leading to the fabrication of a material and it implies the design, the preparation and the characterization of crystalline species.

In this context, the accurate analysis of those linkages that build up the desired structural motifs, are extremely important. Most of these bonds are, however, more elusive than typical chemical bonds of organic molecules, whose nature is known and well rationalized since decades.

Coordinative bonds in metal organic frameworks are most of the time well known because identical to those typical of simple complexes and often understood within the ligand field theory [4]. On the other hand, it is the regio-selectivity in multi-dentate organic linkers to be more intriguing and sometimes difficult to predict.

Even more complicated is understanding the nature and the role of various intermolecular non-covalent interactions in crystals based only on weaker forces, see Table 1 for a summary. This field has attracted enormous attention, starting from the most well-known of these interactions, namely the hydrogen bond [5] (HB).

**Table 1:** **Overview of the most important interactions occurring between two c** **losed-shell electron density distributions (R is the distance between the two centers of masses)**

Interaction type	Origin	Range	Directionality	Contribution
*Electrostatic*	Coulomb attraction/repulsion between unperturbed electron densities	Long range especially monopolar charges of ions (÷1/R^L+1^; where L is the sum of the multipole orders; L = 0 for charge-charge interactions)	Monopole-monopole interactions are not directional; increasing directionality for higher multipolar moments	Stabilization or destabilization, depending on the sign and orientation of the electrostatic moments of the interacting systems
*Induced polarization*	Coulomb attraction between electron density of one molecule and field induced polarization of the other	Shorter range (÷1/R^4^)	Medium-Small	Stabilization
*Dispersion*	Coulomb attraction between mutually polarized electron densities	Quite short range (÷1/R^6^)	Small	Stabilization
*Short range repulsion*	The reduced probability of having two electrons with the same spin very close to each other (Fermi-hole)	Very short range (÷1/R^12^ or exponential)	None	Destabilization
*Charge Transfer*	Interaction between frontier orbitals of the interacting systems it implies partial covalence	Occurs only for contacts shorter than van der Waals distances	Very high	Stabilization

Recognition and classification of intermolecular bonding features is important not only to understand the key factors that promote aggregation, but also to enable the classification of solids through topological analysis [6], which is a method to rationalize both the structural motifs and, at least in principle, the resulting material properties, thus the fundamental steps of a proper material design.

Since the early days of X-ray diffraction, it became clear that it was in principle possible not only to ascertain the positions of atoms in crystals, but also to observe the distribution of electrons [7] and therefore to “visualize” the chemical bonding. This became really feasible much later [8] and it is nowadays quite common to analyze molecules and crystals in terms of electron density partitioning [9]. Among the most relevant achievements, important is the analysis of chemical bonding, through the quantum theory of atoms in molecules (QTAIM) [10], which has been successfully applied to coordinative bonds [11],[12] as well as to most of the known intermolecular interactions [13]–[16]. Moreover, electron density partitioning enables the evaluation of electrostatic interactions between molecules, therefore provides quantitative measures of involved energies.

Methods to obtain the electron density experimentally or to calculate it by first principles are well known and explained in textbooks [9],[17] and review articles [18],[19] and we will not focus on that in this paper.

Here it is important to recall the following concepts and notions:

- The electron density (ED, *ρ*(**r**)) is a quantum mechanical observable, that can be measured, for example, through scattering experiments, in particular X-ray diffraction from crystals. Although it can be directly calculated by Fourier summations of structure factors, the electron density is better obtained as a three-dimensional function fitted against the measured structure factors, which enables a deconvolution of the atomic thermal motion from the (static) electron density distribution.
- The most adopted method to reconstruct the electron density is the multipolar model [20],[21], where *ρ*(**r**) is expanded into atomic - or better pseudo-atomic - multipolar functions, based on a radial function centered at the nuclear site and an angular function (spherical harmonics, usually truncated at hexadecapolar level).
- While the multipolar electron density is not a true quantum mechanical function, it can be compared to those computed *ab initio* by quantum chemical methods that use various degrees of approximation to solve the Schrödinger equation.
  From the electron density some important properties are straightforwardly calculated, like the electrostatic potential, field and field gradients or the electrostatic moments of an atom, a functional group, a molecule or a monomeric unit of a polymer. These partial quantities require that an assumption is made on how to recognize an atom in a crystal (and therefore a functional group or a molecule). The most adopted scheme is offered by QTAIM, but other recent applications make use of Hirshfeld “stockholder” partitioning, as for example the Hirshfeld atom [22] or the Hirshfeld molecular surfaces [23]–[26].
- Other important properties cannot be obtained from the electron density, because they would require not only the trace of the first order density matrix (from which the Bragg scattering of X-rays depends), but also the out of diagonal component or the second order density matrix. These quantities, albeit connected to observables and experimentally available quantities, are very difficult to measure and more often they are obtained only *via* theoretical calculations.
- In the past two decades, methods have been proposed that directly refine elements of density matrices or coefficients of a quantum mechanical wavefunction, including information from scattering experiments (X-ray diffraction, Compton scattering, polarized neutron scattering), see [27] for a comprehensive review on the subject. These approaches, albeit less straightforward than the traditional multipolar expansion, are extremely appealing because they combine theory and experiment and offer a wider spectrum of properties, because the full density matrix becomes available.

In this paper, we present some of the many tools offered by electron density analysis for crystal engineering studies and we will show some applications reported so far in the literature, giving some perspectives for future developments in this field.

## Review

### Electron densities of studies of organic crystals

#### Characterization of Intra- and Inter-molecular interactions

As introduced above, at the basis of crystal engineering is the understanding how molecules interact with each other to form a three-dimensional structure in the solid state. The more insight we get into the nature of these weaker, intermolecular bonding, the more effective materials we can obtain.

With no doubts, QTAIM is one of the most powerful tool for evaluating the interactions within crystal structures, because it analyzes the gradient field of the electron density, hence it enables visualizing its concentrations and depletions, knowing that electrons are in fact the “glue” that stick atoms and then molecules together. QTAIM is grounded on the idea that atoms can still be identified in molecules and provides the quantum mechanical bases for that [10],[28]. This justifies the hard space partitioning of *ρ*(**r**) into atomic basins (Ω) used to quantify atomic volumes and electron populations. The inter-atomic surface (IAS) shared by two bonded atoms enables to evaluate the nature of the bonding between them, especially analyzing the electron density properties at the bond critical point (BCP), a point on IAS where the gradient of ED is equal to zero (∇*ρ*(***r*** ) = 0). In order to extract chemical information on the bond, such as its strength, order, polarity etc., properties evaluated at BCPs are crucial. One of the most important electronic property at BCP is the Laplacian of electron density, ∇^2^*ρ*(***r*** ). Bader *et al.* [29] noted that covalent bonds are typically associated with the approach of the valence shell charge concentrations of the bonded atoms, producing a local accumulation of charge at the BCP, thus characterized by a negative ∇^2^*ρ*(***r*** ). On the contrary, a positive Laplacian indicates the local depletion of electron density, typical of closed-shell interactions, *i.e.* interaction between two electronic systems with the outermost electronic shell filled, as it occurs in ionic bonds, or any other interaction between molecules (van der Waals, medium-weak hydrogen bonding etc.).

**Figure 1:**
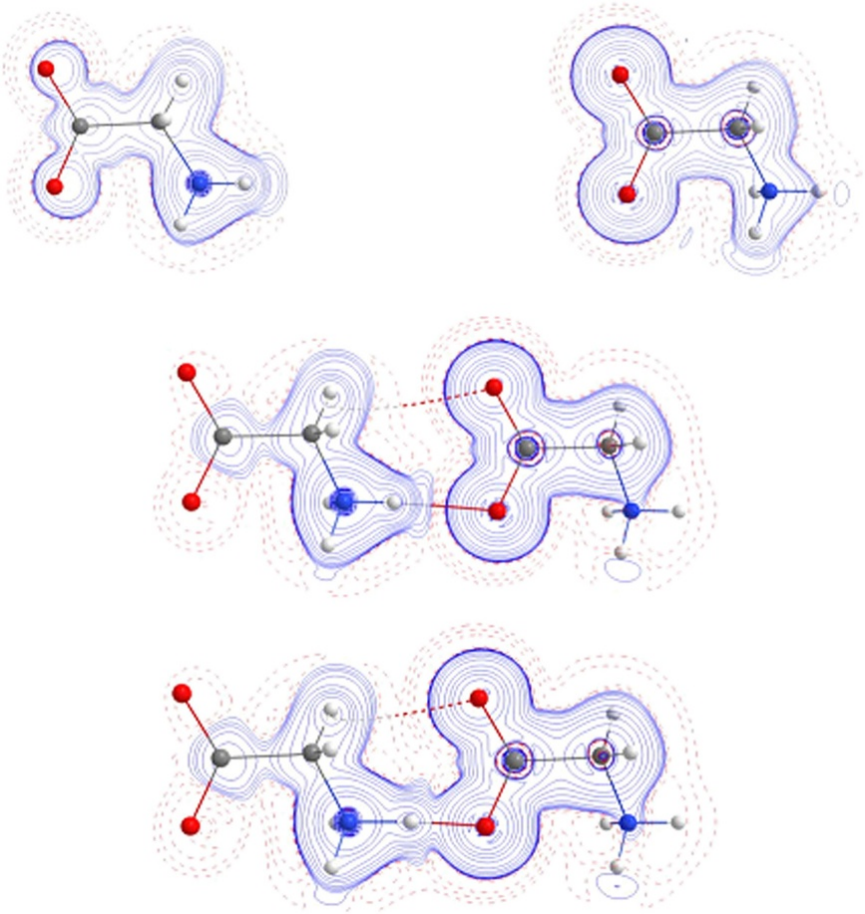
**Energy density distribution** ***H*** **(r) (blue solid lines for negative values; red dashes for positive values) for two glycine zwitterions approaching to form a strong hydrogen bond.** The plots are drawn in the plane containing the carboxylic group of the HB acceptor molecule: top, long distance between donor and acceptor atoms (8 Å); center the equilibrium position in the crystal (2.76 Å); bottom, very short distance as in symmetric hydrogen bonds (2.4 Å). Note that *H*(**r**) has uninterrupted regions of negative values in the bottom plot. A weaker C − H^…^O bond path is also calculated (dashed bond path).

This paradigm works well for most organic compounds but it fails when heavier atoms (*e.g.* transition metals) are concerned [30]. In fact, the rather elusive outermost shell of these elements, makes the sign of ∇^2^*ρ*(***r*** ) no longer discriminating. For almost all bonds to a transition metal, the corresponding BCPs are found in regions of charge depletion [11],[12], thus producing a kind of “Hegelian night”. For this reason, other indicators were found to be more useful, for example the energy densities and the electron delocalization indices that however require the entire first order density matrix to be calculated, therefore they cannot be retrieved just from the electron density (trace of the first order density matrix), which is a more straightforward observable. A local kinetic *G*(***r*** ) and potential *V*(***r*** ) energy density functions can be defined from the first order density matrix. Cremer and Kraka [31] were the first to introduce the idea that the total energy density *H*(***r*** ) (=*G*(***r*** ) + *V*(**r**)) reflects a dominant covalence when *H*(***r*** ) <0 (*i.e.* when the potential energy density is in excess). As a matter of fact, the total energy density, better than the Laplacian, defines sensible boarders of a molecule, see for example Figure 1, in which the *H*(***r*** ) distribution of two approaching glycine molecules is drawn. When a strong hydrogen bond is eventually formed, the valence regions of the two molecules belong to the same synaptic domain of negative energy density, thus *H*(***r*** ) at the H^…^O BCP is negative, in keeping with most of the current consensus that for such short distances the bond must contain significant amount of covalence. Electron delocalization indices, instead, measure the number of electron pairs shared by two atomic basins [32] and can also be used to reveal the degree of covalence in intermolecular bonding. In addition, they may anticipate exchange paths in magnetic frameworks, especially in metal based materials. The nature of metal-ligand bonds is extremely important in one sector of crystal engineering, namely that of coordination polymers, and will be discussed in section 3. Other interactions between molecules are more genuinely classified into the closed-shell class, although some amount of covalence might be present, sometime playing a fundamental role.

In order to characterize genuine hydrogen bonds and to differentiate them from pure van der Waals interactions, Koch and Popelier [33],[34] proposed eight conditions to be fulfilled. The first four criteria can be easily checked also from experimental data, because relying only on the electron density function and its derivatives in the crystal, whereas others comparisons of quantum mechanical calculations for the HB aggregate and the isolated molecules. The presence of a BCP between donor and acceptor atoms linked through a bond path and the presence of charge density at the BCP is the basis of first two criteria. Positive value of the Laplacian at BCP and its correlation with interaction energies constitutes the third condition. Noteworthy, this condition can be controversial because very strong, symmetric HB’s are associated with negative Laplacian, indicating even large stabilization energy. The fourth criterion, considered as “necessary and sufficient”, concerns the mutual penetration of the hydrogen (H) and the acceptor (A) atomic basins. The following relation must be fulfilled:

1 $$\Delta r_{H}+∆r_{A}=\left(r_{H}^{0}-r_{H}\right)+\left(r_{A}^{0}-r_{A}\right)>0$$

where $r_{H}^{0}\text{,}$$r_{A}^{0}$ are non-bonded radii of hydrogen and the acceptor atom taken as the gas phase van der Waals radii and *r*_*H*_, *r*_*A*_ are corresponding bonding radii taken as distances from BCP to the nuclei. Any violation of the above condition indicates van der Waals nature of the considered contact. Other criteria express a loss of electrons and energetic destabilization of H-atom resulting from increased net charge of the atom, as well as a decrease of dipolar polarization and volume depletion of H-atom.

Another convenient classification of weak electrostatic interactions is based on the electronic energy densities, introduced by Espinosa *et al.* [35], who extended the idea by Cremer and Kraka. In fact, weak electrostatic interactions, can be classified in terms of kinetic energy density *G*(***r***_BCP_) and potential energy density *V*(***r***_BCP_) at the bond critical point. The relationship between those two functions reflects how electrons around BCPs are affected by the formation of a hydrogen bond (HB). As mentioned above, energy densities in principle require the full density matrix to be computed, however Abramov [36] proposed a functional to estimate the kinetic energy density, based only on the electron density and its derivatives, therefore making it available to experimental determinations as well. In particular, at points where ∇*ρ*(***r*** ) vanishes (like all the critical points of *ρ*(***r*** ), CP’s):

2 $$G\left(r_{CP}\right)=\frac{3}{10}{\left(3\pi^{2}\right)}^{2/3}\rho^{5/3}\left(r_{CP}\right)+\frac{1}{6}\nabla^{2}\rho \left(r_{CP}\right)$$

In turn, the potential energy density *V*(***r***_CP_) is then obtained applying the local virial theorem:

3 $$V\left(r_{CP}\right)=\frac{\hbar^{2}}{4m}\nabla^{2}\rho \left(r_{CP}\right)-2G\left(r_{CP}\right)$$

Following Cremer and Kraka, in closed-shell interactions, the local kinetic energy density *G*(***r***_CP_) (everywhere positive) is in excess of local potential energy density *V*(***r***_CP_) (everywhere negative), thus H(***r***_CP_) >0. Furthermore, the larger is |*V*(***r***_CP_)∣, the larger is the shared character of the interaction and the electronic stabilization of the structure. It is also observed that in closed-shell interactions the amount of kinetic energy per electron is large, typically *G*(***r***_CP_)/*ρ*(***r***_CP_) >1 (in atomic units). Because at BCP the kinetic and potential energy densities depend exponentially on the distance between hydrogen atom and the HB acceptor, a correlation was found between the energy of the hydrogen bond and potential energy density [35]:

4 $$E_{HB}=\frac{1}{2}V\left(r_{CP}\right)$$

which can be interpreted as the energetic response of the hydrogen bond to the force exerted on the electrons around BCPs. Note that the ½ coefficient in equation (4) is not dimensionless. Spackman [37] has shown that this correlation can be actually predicted even from the *pro-crystal* ED, *i.e.* from the simple summation of atomic spherical electron density functions that are easily calculated once the crystal structure is known.

The above mentioned quantitative indicators can be used to analyze any weak interactions and were extensively applied by Munshi and Guru Row [38]. They first reported on a comparison between experimental and theoretical electron density studies for three bioactive molecules: 2-thiouracil, cytosine monohydrate and salicylic acid. They gave a quantitative description of all the interactions and could clearly differentiate strong and weak contacts. Moreover, they showed that the nature of weak interactions is not lost in the presence of strong hydrogen bonds. Those studies contributed to evaluation of preferred orientations at the protein binding sites. The same group studied for the first time the differences of the electron density in polymorphs of 3-acetylcoumarin [39]. This research clearly indicated that for the purpose of “quantitative crystal engineering” conventional crystal structure analysis based only on geometrical features is insufficient and inadequate. Only detailed ED analysis can justify the occurrence of any interaction in the crystalline state and therefore provide a useful input to design new materials. Extensive studies on aliphatic dicarboxylic acids [40] revealed interesting systematics in the topology of ED. The electron density associated with the side-chain interactions, as a fraction of the total intermolecular density, plotted against the number of methylene groups revealed an alternating behavior. Acids with even numbers of carbons exhibit higher ρ(r) values at bond critical points, compared to their odd neighbors. This explains the relatively higher melting points in the even-member acids since side-chain interactions play a major role for the cohesion of acid molecules in the solid state. Howard and co-workers [41], based on experimental ED studies of *trans-*cinnamic acid and coumarin-3-carboxylic acid, postulated that the presence of strong interactions not maintained in the irradation products “may influence the ability of a compound to undergo a solid-state [2,+2] cycloaddition reaction”.

A general hypothesis concerning azide building blocks was proposed by Bushmarinov *et al*. [42]. Based on the QTAIM and the electron localization function (ELF [43],[44]), geometrical preferences in favor of hydrogen bond formation were explained. They proved that the number of interactions to the terminal nitrogen atoms of the azide only depends on steric effects, thus supramolecular systems based on hydrogen bonds to an azide will be independent from the torsions involving terminal atoms of the azide.

Important insight into the understanding the crystal structure –property correlation was given by Gopalan *et al.* [45]. They studied organic crystals exhibiting NLO properties and showed how the non-centric nature of the crystal field affects molecular dipole moment and therefore optical properties of the solid.

Increasing attention is attracted by halogen bonded [46]–[48] crystals. In terms of charge density analysis, Bianchi *et al.* [49] reported on the investigation of the co-crystal of 1,2-bis(4-pyridil)ethylene with 1,4-diiodotetrafluorobenzene. Based on QTAIM topology, they classified the interaction between the pyridyl donor N and the di-iodobenzene I acceptor as a closed-shell interaction. Indeed this is characterized by a positive Laplacian at the intermolecular BCP, although accompanied by a negative energy density as the authors also pointed out. A clear manifestation of the “Hegelian night” is that C-I interactions would appear, at first sight, “similar to those of metal-metal and metal-ligand bonds in organometallic compounds” [49]. Interestingly, the authors proved that equation (4) remains substantially valid for this interaction, by comparing the *ab initio* interaction energies with the empirical derivation from the kinetic energy density.

Bui *et al.* [50] developed a model to rationalize halogen bonding based on accurate studies on hexa-halobenzene molecular crystals. The deformation density, *i.e.* the difference between the total electron density and the superposition of spherical atomic densities (hereinafter called the *promolecule*), enabled the visualization of the so-called *σ-hole*, first anticipated by Politzer *et al.* [51],[52] using quantum chemical calculations. Other studies have followed [53]–[55], again stressing on the visualization of the *σ-hole* by means of the electrostatic potential or the Laplacian distribution, and therefore addressing the overwhelming contribution of the electrostatic term. However, recent work by Stone [56] has demonstrated that some stereochemical features of the halogen-bonded packing originate from the necessity to minimize the inter-atomic repulsion term, rather than from a stabilizing, though weak, electrostatic interaction. Accordingly, Spackman has very recently shown that in many cases the interaction between halogen bonded molecules is associated only with a small or even negligible stabilization [57]. Therefore, further investigations are expected in the next future on this topic.

To facilitate the discussion of all intermolecular contacts in molecular crystals it is very useful to introduce Hirshfeld surface (HS) analysis [23]–[26]. The Hirshfeld molecule is an extension of the concept of Hirshfeld atom [22], which is not based on a quantum mechanical definition, as QTAIM, but on a rather simplified interpretation of a multivariate function, like the electron density when a breakdown into atomic terms is adopted. Hirshfeld defined the atom as a “stockholder”, who receives from the “asset” an “equity” proportional to the “investment”. In this *naive* example, the asset is the molecular electron density (computed or measured), whereas the investment is the electron density of the isolated atom, calculated in its ground state and spherically averaged. The equity, evaluated at each point and integrated over whole space, can be positive or negative, leading to a negatively or positively charged atom respectively ^a^.

**Figure 2:**
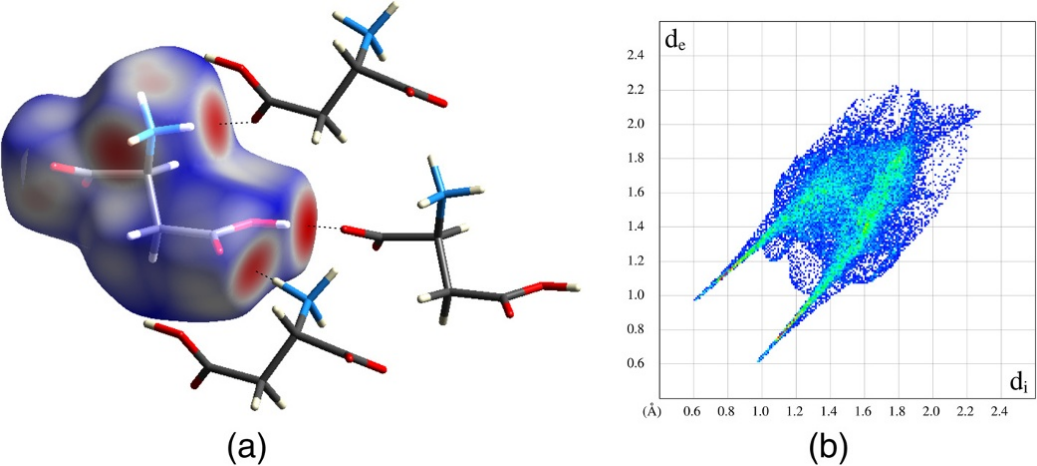
**Hirshfeld surfaces and fingerprint plots. (a)** Hirshfeld surface of L-aspartic acid with d_norm_ plotted from -0.799 (red) to 0.976 (blue) Å. The volume inside the HS is 128 Å^3^. **(b)** 2D fingerprint plot. Drawings plotted using CrystalExplorer [59].

In case of a molecule in a crystal, *mutatis mutandis*, the same concept can be applied. However, Spackman realized that the fuzzy partitioning of the Hirshfeld approach (each point in space belongs to many atoms, with its own share) was not very useful for crystal engineering. A hard space definition of the building blocks is much preferable. Therefore, he defined a molecule in a crystal as the, unique, region of space whose *procrystal* density has at least 50% share from the given pro-molecule. Noteworthy, a tessellation of space is not complete with this partitioning, because regions without a dominant pro-molecule are in principle possible, albeit in general extremely small. The Hirshfeld surface gives a unique signature of a molecule in a crystal, because it strongly depends on the surrounding, so the same molecule in different crystal packing looks different. On the Hirshfeld surface, some functions can be mapped, as for example d_*norm*_, which combines the internal d_i_ and external d_e_ distances from the surface to the nearest nucleus. On Figure 2a, the Hirshfeld surface of L-aspartic acid (L-Asp) is shown: contact zones shorter than van der Waals radii are marked as red areas and highlight hydrogen bond sites of the molecule. Hirshfeld surfaces are very often accompanied by 2D fingerprints [58],[59], Figure 2b, scatter-plots of d_e_ and d_i_ that uniquely identify each type of interaction in the crystal structure. In case of L-Asp, the strongest interactions are those of O…H type constituting the highest fraction of 72.7%. Other close contacts are also present, including very weak C…H interactions (2.8%) and non-directional H…H contacts contributing in 18.9%.

Beside the numerous applications of this methodology and its growing appeal in crystal engineering, it should be stressed that this analysis does not rely on quantum mechanics and therefore its predictive power is based only on empirical evidences.

A recent and alternative way of quantifying non-covalent interactions (NCIs) between molecules was introduced by Johnson *et al.* [60] and Contreras-García *et al.* [61]. The NCI descriptor enables visualizing regions of space involved in either attractive or repulsive interactions. The NCI index depends on the reduced electron density gradient (RDG):

5 $$s\left(r\right)=\frac{\left|\nabla \rho \left(r\right)\right|}{2{\left(3\pi \right)}^{1/3}\rho {\left(r\right)}^{4/3}}$$

Scatterplots of *s*(**r**) against *ρ*(**r**) address non-covalent interactions. In fact, in the low-gradient and low-density regions characteristic spikes occur which are not observed for covalent bonds. If we only consider ED/RDG regions, the information about the nature of the interaction would be lost, since different types of interactions appear in the same very narrow range. However, the sign of the second eigenvalue *λ*_2_ of the Hessian matrix of *ρ*(**r**) (with ∇^2^*ρ*(***r*** ) = *λ*_1_ + *λ*_2_ + *λ*_3_); *λ*_1_ ≤ *λ*_2_ ≤ *λ*_3_) indicates whether the interaction is stabilizing (*λ*_2_ < 0) or destabilizing (*λ*_2_ > 0). Therefore, diagrams of *s*(*ρ*(**r**) ⋅ sign(*λ*_2_)) allow recognizing the type of NCI, whereas the amount of density itself issues the strength of that interaction. A spike in the low-gradient, low-density area at negative *λ*_2_ indicates stabilizing interactions like hydrogen bonds, a smaller spike and slightly negative *λ*_2_ is the fingerprint of a weaker stabilizing interaction, and a spike associated with positive *λ*_2_ indicates that no NCI is active. The shape of RDG surfaces also allows for qualitative description of interactions strength. Small disc-shaped RDG domains denote stronger interactions whereas broad multiform domains refer to much weaker interactions. NCI approach can be applied to experimental or theoretical electron density distributions, as for example shown by Saleh *et al.* [62] or by Hey *et al.* [63].

The importance of intermolecular interactions can be evaluated also through the analysis of atomic polarizabilities, in particular their deformation with respect to non-interacting molecules. Recently, we have developed a program, PolaBer [64], which enables to calculate distributed atomic polarizabilities based on a partitioning of the electron density. The advantage of this approach is the definition of atomic contribution to a molecular property (the molecular polarizability) or a crystalline property (the linear susceptibility), which enables to identify the key-features for large polarizabilities. Therefore, this approach might be useful for crystal engineering purposes.

**Figure 3:**
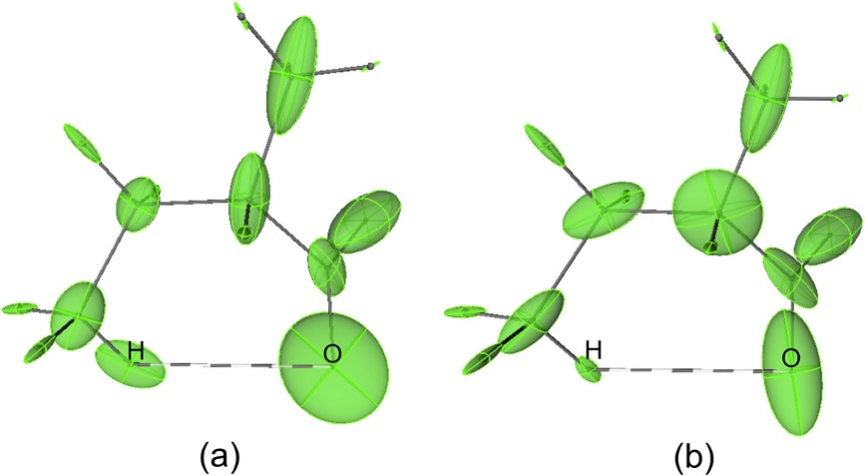
**Graphical representations of atomic polarizabilities in L-valine with different treatment of weak intramolecular interaction C-H…O: (a) no weighting scheme applied, no distinction between the strength of bonds is taken into account (b) weighting scheme applied.** Atomic polarizabilities are drawn with a scaling factor of 0.4Å^−2^.

The ED partitioning follows QTAIM, although other schemes could be adopted. The main advantage of QTAIM is that it is based on quantum mechanical ground, therefore together with atoms in molecules one consistently define bonds as well. Moreover, it ensures a maximal transferability between different systems as already demonstrated by Matta and Bader [65]. Within this approach atomic properties such as charges *Q*(Ω), energies E(Ω) and, in particular, dipole moments *μ*(Ω) can be calculated by integrating their corresponding operators over the volume of atomic basin Ω. Atomic polarizability tensors are obtained from numerical derivatives of atomic dipole moments with respect to external electric field:

6 $$\alpha_{ij}\left(\Omega \right)=\frac{\mu_{i}^{\varepsilon_{j}}\left(\Omega \right)-\mu_{i}^{0}\left(\Omega \right)}{\varepsilon_{j}}$$

where $\mu_{i}^{F_{j}}\left(\Omega \right)$ is the atomic dipolar component along the *i* direction computed with a given electric field (0 or ε) in *j* direction. Full description of the procedure is given in Krawczuk et al. [66], based on the theory developed by Keith [67]. For crystal engineering purposes, it is essential to differentiate weak non-covalent intra-molecular or intermolecular interactions from covalent bonds inside the molecule. In fact, the partitioning scheme distinguishes two contributions to the atomic dipole: one is due to the polarization inside the atomic basin, the other originates from distributing the atomic charge over all the bonds to the atom creating a bond dipole. These quantities are easily computed from a system of equations involving all bonds and all atomic charges, however an ambiguity occurs when a ring is present. Keith [67] suggested including an additional condition to enable solution of the system of equations: the sum of ring bond charges should be zero. However, if all bonds are taken as equivalent in the ring, an anomalous importance is attributed to weaker interactions, producing mathematically correct but physically unrealistic atomic polarizabilities. Therefore, a weighting scheme is applied in PolaBer: in the ring conditions, bond dipoles are multiplied to the inverse of their strength, measured by the electron density at the BCP. This avoid drastic changes of the atomic polarizabilities, if a weak BCP generates a ring in the molecular graph. On the other hand, rings made of strong covalent bonds (like those of aryls) truly affect the atomic polarizabilities; accordingly all bonds have similar or even identical weight if symmetric. On Figure 3 atomic polarizabilities in L-valine are visualized. In the zwitterionic form, an intramolecular weak hydrogen bond of C − H…O type is present. If no weighting scheme is applied, the polarizabilities of oxygen and hydrogen atoms are substantially different than those of the same molecule in a conformation where no intramolecular HB occurs.

**Figure 4:**
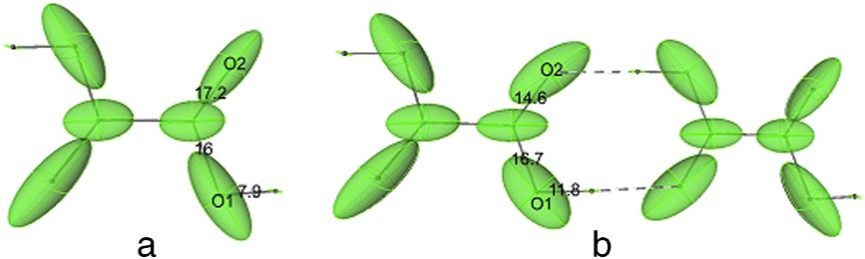
**Atomic polarizability ellipsoids for (a) isolated oxalic acid molecule and (b) oxalic dimer bounded by O-H…O hydrogen bond.** Note that the size and orientation of O1 and O2 ellipsoids change when a HB is formed. Scaling factor of polarizabilities is 0.4Å^−2^.

Since atomic polarizabilities are second order positive tensors, they are easily visualized as ellipsoids with main axes having dimensions of volumes. The visualization is done in the same real space as the molecule assuming that 1Å^3^ (unit of polarizability tensor) =1Å (unit of atomic coordinates), though for visualization purposes a scaling factor is necessary to reduce the size of ellipsoids (typically 1Å^3^ = 0.4Å for atoms of the second period). The size of the ellipsoid is proportional to the total atomic polarizability, whereas the ellipsoid axes indicate the anisotropy of the polarizability, thus the directions along which the atomic electron density is more or less polarizable. Although weaker than covalent bonds, hydrogen bonds may affect the polarizabilities. The perturbation is mainly due to the electrostatic interaction occurring between the donor and the acceptor atoms in the hydrogen bond system. On Figure 4, a comparison between isolated molecule of oxalic acid and a dimer is shown. In general, polarizabilities are larger along covalent bond directions, especially towards atoms with high polarizabilities (see carboxylic groups). When a hydrogen bond is formed, the oxygen atoms are slightly modified in orientation and are stretched along HB direction (compare O1 and O2 ellipsoids in both pictures), due to the perturbation produced by the incoming donor atom. The increased polarizability along the direction of the HB can be measured by the *bond polarizability* defined as the projection of the atomic ellipsoids on the bond vector:

7 $$\alpha_{\Omega -\Omega '}\;=\;r_{\Omega \Omega '}^{T}\cdot \left(\alpha_{\Omega }\;+\;\alpha_{\Omega '}\right)\cdot r_{\Omega \Omega '}$$

where **r**_***ΩΩ***'_ is a unit vector in the direction of **Ω**-**Ω’** bond. *α*_***Ω*** − ***Ω*** Ω'_ is a scalar which reflects how feasible is the polarization of the electron density along the bond, upon application of an electric field in the same direction. Values of bond polarizabilities for carboxylic groups of oxalic acid are also given on Figure 4. Larger values of bond polarizabilities of O-H bond in dimer confirm the elongation of hydrogen polarizability along the donor-acceptor path.

#### Co-crystals

The design of co-crystals for multifunctional materials has brought lots of attention in last few years, especially in the field of pharmaceutical compounds [68]–[71] where at least one of the components is an active pharmaceutical ingredient (API). So obtained co-crystals gain new chemical and physical properties (*i.e.* solubility, density, hygroscopic abilities, melting point etc.), usually drastically different from individual components and ideally tunable in order to obtain the desired functionality. The crucial point in crystal engineering of drugs is to understand and evaluate potential intermolecular interactions that a given molecule may exhibit and rationalize the consequences for the supramolecular architecture.

One of the first charge density analysis on API co-crystals was presented by Hathwar and co-workers [72]. The main goal of the study was to quantitatively describe differences between a co-crystal of nicotinamide (API component) with salicilic acid and the salt formed by nicotinamide and oxalic acid. The region of main interest was the proton transfer path to the nitrogen atom on the pyridine ring of nicotinamide. Topological analysis revealed bonding features associated with N…H − O and N − H…O hydrogen bonds for the salt and the co-crystal, respectively. A similar picture was obtained from the electrostatic potential maps where the electropositive region on oxygen atom of salicilic acid indicated close-shelled interaction whereas electronegative region of oxygen atom on oxalic acid suggested covalent bond with H atom. All above observations confirmed earlier hypothesis that a formation of a co-crystal is strongly dependent on pK_a_ of the individual constituents. Those studies offered very convenient way of verifying the continuum from co-crystal to salt by assessing interaction energies in terms of charge transfer character at the critical point.

Hathwar *et al.* [73] proposed also a library of transferable multipolar parameters for structural fragments representing supramolecular synthons. Based on the high resolution X-ray diffraction datasets, the library would provide the criteria to design and fabricate new synthons and therefore mimic the 3D formation based on a given hydrogen bond system. Since it was already proven that multipolar parameters are transferable for molecules or molecular fragments [74]–[82] authors wanted to test if this is also true for supramolecular synthons. The transferability was tested on methoxy-benzoic acid, acetanilide, 4-(acetylamino)benzoic acid, 4-methylbenzoic acid, and 4-methylacetanilide. Electron density features derived with the supramolecular synthon based fragments approach (SBFA), were compared to experimentally obtained values and showed a very good agreement, except for some discrepancies in monopole parameters. The SBFA can be successfully applied for essential topological features of ED for intra- and intermolecular interactions (synthons) in molecular crystals, especially when no good quality crystals can be obtained and therefore no high-resolution data can be gathered. SBFA model was also applied for synthons with C − H…O, C − H…F and F…F interactions [83] proving that this model can also be applied for weak interactions.

Charge density studies provide valuable information on subtle features in case of polymorphism in co-crystals. In our best knowledge, so far only couple of papers reported on charge density studies for polymorphs of co-crystals. Gryl *et al.* [84] confirmed earlier hypothesis [85] that the polymorphic forms of barbituric acid and urea originate from the existence of resonance structures of the barbituric acid molecule. Both, experimental and theoretical charge density studies indicated characteristic features of two, among six, possible mesomeric forms, see Scheme one in [84]. It was possible to recognize electron density displacement in barbituric acid molecule towards those two resonant forms, which influence the type of hydrogen bonds formed in each polymorphic form and therefore results in different packing topology.

Schmidtmann et al. [86] studied short, strong hydrogen bonds (SSHB) of O − H…N type in two polymorphic forms of isonicotinamide-oxalic acid crystallizing in C 2/c (I) and P‾1 (II) space groups. It was the first case where topological analysis of ED confirmed formation of rather unusual centered heteronuclear intermolecular SSHB of O…H…N type between oxalic acid and isonicotinamide in polymorph II. The presence of such an interaction, where carboxylic H atom is equally shared between O and N atom, raises the question whether this compound should be considered as a co-crystal or a salt, however this is beyond the scope of this paper.

Dubey *et al.* [87] applied supramolecular synthon based fragments approach [72] to study polymorphism of orcinol:4,4′-bipyridine co-crystals and showed the transferability of multipole parameters of O − H…N synthon in the polymorphic forms of the studied compound.

Although a rather small number of studies have reported on co-crystals from charge density point of view, all above examples clearly indicate the importance of the topology of electron density towards understanding the correlation between crystal structure and the physical property of the materials.

#### Optical properties from electron density studies

One of the most challenging part of crystal engineering is to design an efficient material which will exhibit desired physical properties. To accomplish this goal, a deep understanding of the connection between molecular structure and the solid-state property is needed. In this chapter we want to focus on optical properties and attempt to estimate those properties from electron density studies, both experimental and theoretical.

The optical response of the material is determined by its electric susceptibilities, that depends on the dipole (hyper)polarizabilities of individual atoms and therefore molecules. The electric (hyper)polarizabilities of a molecule have long been used to predict and understand their chemical reactivity, intermolecular interactions and physical properties. For example, the first order polarizabilities enable to calculate refractive indices, applying the Clausius-Mossotti equation [88],[89] or, taking into account long range interactions, the anisotropic Lorentz field factor approach [90].

There were several attempts to obtain molecular (hyper)polarizabilities from electric moments using experimental charge density studies of materials with potential non-linear optical properties and therefore to predict the size of the NLO effect. Within first reports [91]–[93], the estimation of NLO properties was done from a Robinson model [94] allowing to connect molecular polarizabilities to multipolar moments of the electron density distribution. First results were very promising, especially in case of molecular polarizability, but failed when estimating the hyperpolarizabilities. Explanation for this was given later by Whitten *et al.* [95] who stated that the electron density obtained from regular multipole model does not carry sufficient information to determine reliably molecular hyperpolarizability and therefore to estimate correctly the non-linear optical effect. Instead, the X-ray constrained wavefunction (XCW, [96]) approach, implemented in the software package TONTO [97], provides a *pseudo-*quantum mechanical wavefunction constrained to reproduce the experimentally observed structure factors. The wavefunction calculations minimize the sum of Hartee-Fock energy and the χ^2^ function which defines the precision level of the experimental structure factor against the calculated ones:

8 $$\chi^{2}=\frac{1}{N_{r}-N_{p}}\sum_{h}^{N_{r}}\frac{{\left[F\left(h\right)-F^{*}\left(h\right)\right]}^{2}}{\sigma^{2}\left(h\right)}$$

where *N*_*r*_ and *N*_*p*_ are numbers of reflections and parameters, respectively, *F* and F^*^ are calculated and experimental structure factors and *σ*^*2*^ is the uncertainty of each structure factor. This leads to an “experimental wavefunction” from which physical properties can be easily calculated, thanks to the fact that a *pseudo* density matrix can be defined. In a recent paper [98], the XCW model was successfully applied to calculate molecular dipole moments, polarizabilities and refractive indices of small organic compounds (which find application in laser dyes) using coupled perturbed Hartree-Fock (CPHF) approach, therefore calculating the field induced perturbation to the molecule embedded in the crystal. A new XCW approach was recently proposed by Genoni [99], who implemented the method for extremely localized molecular orbital wave functions. An alternative way to estimate the crystal properties is however that of calculating with high accuracy the molecular quantities, then applying corrections for the perturbation of the crystal packing. For example, quite effective is the distributed atomic polarizability approach to estimate linear optical properties implemented in PolaBer [89]. The atomic polarizabilities can be used to calculate the electric susceptibility, through the anisotropic Lorentz approximation. The advantage of using atomic polarizabilities rather than the whole molecular ones, is that we can extract separate information about the role of each functional group in the molecule, which is very important to design new molecules. One nice example is the calculations of refractive indices recently reported for the L-histidinium hydrogen oxalate crystal structure [100]. Obtained values of refractive indices were comparable with the ones obtained from couple-perturbed Kohn-Sham theory, although slightly different from the experimental ones. In this kind of comparison, one should consider that calculations are generally carried out at zero frequency instead of finite one, and therefore refractive indices are underestimated. Nonetheless, these results are promising and could open a new field in applications of electron density partitioning for material properties.

## Metal organic materials

As discussed in the previous sections, the electron density analysis offers many tools to investigate materials, in particular the stereo-electronic features that enable understanding the robustness of a given type of aggregation or the breakdown of a crystal property in terms of atoms, functional groups or molecular building blocks.

For metal organic materials, and in particular porous metal organic frameworks (MOFs) [101], quite useful is the possibility to “observe” interactions occurring in cavities, channels or layers, where guest molecules or counter ions can be hosted and could diffuse, for example during ion exchange processes.

For example, Hirshfeld surfaces have been adopted not only to define molecules in crystals, but also as a qualitative tool to investigate mutual relationships between building blocks of materials [102] and to find possible exchange channels for ions [103]. In fact, HS could be used, on one hand, to visualize the complementarity between functional groups in building blocks and therefore to visually address potentially robust synthons. This relatively simple approach has been enthusiastically received in the crystal engineering community, so that HS plots usually accompany many papers in this field, although, as already discussed, the quantum mechanical information therein is sometime overestimated. On the other hand, the *procrystal* electron densities enable the visualization of sites available for guest molecules and therefore potentially usable channels in porous frameworks. An available site is expected when the *procrystal* electron density is below a given threshold. Albeit heuristic, this concept implies that a guest molecule can be hosted in a framework only in regions where the short range repulsion associated with the Pauli exclusion-principle (Table 1) is small enough. In fact, it is demonstrated that this repulsion is proportional to the overlapping density [104], therefore a region of small electron density of the framework should be more accessible. Moreover, as discussed in section 2, the electron density is proportional to the amount of kinetic energy density, which would produce destabilization, therefore the criterion is actually grounded on energy considerations.

This qualitative picture calls for more accurate evaluation of the stabilization or destabilization produced when two molecules interact. Since the 1980s’, Spackman [105]–[108] has proposed simple models to evaluate the electrostatic energy of two sets of multipolar distributions, based on the classical point-multipole approach by Buckingham [109], though including corrections for the diffuse nature of the electron density distributions that could also penetrate one into the other. Moreover, he proposed a set of atom based parameters to estimate the repulsion as well as the dispersion. In the classical McWeeny approach [110], the electrostatic energy is simply the zero order energy of the Coulombic interaction between the two electron density distributions, see also Table 1. At this point, it should be reminded that the actually observed electron density in crystals implicitly includes the polarization induced by the electric field experienced by the molecule in the crystal, therefore the electrostatic energy computed from experimentally derived multipolar parameters includes the first order perturbed polarization energy [111]. The dispersion energy, instead, comes from the mutual perturbation of excited configurations and it is often regarded as the electrostatic interaction occurring between instantaneous multipoles of the two interacting systems.

Repulsion and dispersion are normally estimated by atom parameterized functionals, that depends on the n^th^ of the distance between atoms (6th and 12th power for dispersion and repulsion, respectively, in classical Lennard-Jones potential [112]) or otherwise have exponential decay.

After the pioneering work by Spackman, other approaches have been proposed, which are especially trying to overcome the inaccuracy of a point-multipole approximation. For example, Gavezzotti [113] has proposed the well-known PIXEL approach, where the electrostatic interaction is evaluated in terms of Coulomb interactions between the charge of the interacting molecules at each point (pixel) in space. This is nothing else than a numerical solution to the Coulomb integral of two charge distributions, that Volkov and Coppens [114],[115], instead solved with a more refined quadrature, obtaining a considerable speed up in the calculation. The more interesting feature of Gavezzotti’s approach was the estimation of polarization energy, genuinely deconvoluted from zero order electrostatic interaction if starting from molecular electron densities evaluated in the gas phase, as well as the estimation of repulsion and dispersion. Repulsion follows the idea of Wheatly [104], through the evaluating of the overlap integral between two electron density distributions, whereas the functionals for polarization and dispersion [116] make use of the concept of polarizability functions [117]. Thus, polarization is the energy obtained by summing at each point the interaction between the electric field of one molecule and the local field induced dipole of the other which depends on the molecular (or atomic) polarizability. Dispersion instead is the interaction between both polarizabilities, following the original proposal by London [118].

The use of the PIXEL semi-classical sums, although very easy to parallelize and therefore suited for modern GPU’s clusters, is not so convenient for larger systems, where faster algorithms like Volkov’s [114],[115], are more efficient. Moreover, some approximation could be adopted to simplify the calculation: in fact it is not necessary to use the exact electron density functions for molecules or atoms which are quite distant from each other. In this case, the point multipole approximation [109] is sufficiently accurate and much faster to evaluate. For even longer range interaction, the molecular moments, instead of the atomic ones, could be used.

**Figure 5:**
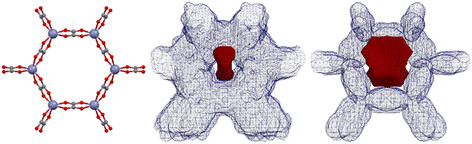
**Left, the idealized structure of Zn oxalate honeycomb framework; center, accessible region (in red) for a large molecular cation like piperazinium [C**_**4**_ **H**_**12**_ **N**_**2**_^**2+**^ **]; right, the accessible region for potassium cation (K** ^**+**^ **).** The blue regions highlight the destabilization, mainly due to short-range repulsion when the cation center of mass is in that site.

Despite the theoretical background is in fact available, interaction energies are rarely used to investigate MOFs or in general of metal organic materials. Adams and Rao [119] proposed an energetic criterion based on Morse-type functionals and the bond-valence mismatch, which seems to be promising and quite simple to use, but limited to inorganic systems. Instead, an interaction energy mapping was proposed by Chimpri and Macchi [120], that while preserving the same simple visualization of threshold density approaches, it contains much more quantitative information because the energy is in fact evaluated in more accurate way using Volkov’s method and therefore it is the total interaction energy to be computed, not only the repulsion. Moreover, the adopted threshold is more physically grounded, because *an available site* is simply defined as *the one for which the interaction energy between the guest molecule and the framework is stabilizing*. It is very important to stress that this energy is calculated from the electron density of the host and the guest, therefore at variance from the criterion of minimal electron density of the host, the interaction energy map is able to screen the capability of the framework to host a particular guest. Some examples of the interaction energy mapping are shown in Figure 5, for some classical metal-oxalate honeycomb structures.

Quite important MOFs are those which maximize the porosity, without losing chemical and mechanical stability. One famous example is MOF5 [121] (Zn_4_O(1,3,5-benzenetribenzoate)_2_), which is often taken as a benchmark for its extraordinary porous properties. Civalleri *et al.* [122] have analyzed the electronic structure and the electrostatic potential *φ*(**r**) in MOF-5, demonstrating the feasibility of a fully *ab initio* simulation and the useful approach based on the electrostatic potential. It is worth pointing out that while the electron density threshold criterion only addresses the short-range repulsion, *φ*(**r**) maps only address the electrostatic attraction/repulsion of a point charge, therefore their interpretation should be considered very carefully.

Among metal framework structures, of particular interest are nowadays those carrying magnetic properties, in particular able to build up 1D, 2D or 3D magnetism. For this reason, ED studies on magnetic frameworks are becoming more common, sometime coupling the electron density with the spin density analysis.

Iversen *et al.* [123]–[125] have deeply investigated the connection between electron density topology and framework structures in magnetic materials, analyzing in particular the stereoelectronics at the metal site. In fact, the multipolar expansion enables to roughly estimate the electronic population of transition metal d-orbitals [126], therefore having at least a confirmation of the electronic structures predicted from the magnetic measurements. In some cases, it is even possible to estimate the magnetic moments [127],[128], by careful consideration of the electronic population. However, the full information on the spin density is available only if using a radiation able to interact with the electronic spin, as for example polarized neutrons [129]–[132]. In particular, recent work by Deutsch *et al.* [133] opens the possibility to simultaneously determine the charge and spin density, from combination of X-ray diffraction and polarized neutron diffraction. Very important is in fact the possibility to determine the paths of magnetic exchange, in view of the growing interest for metal framework materials with tunable magnetism.

## Conclusions

In this mini-review, we have focused on the application of charge density analysis in the vast but still growing field of crystal engineering, showing that crystallographic information, which goes beyond the determination of the molecular geometry or the supramolecular packing, can be of enormous impact for this kind of research.

The charge density is inherently connected with X-ray diffraction, given that it is in fact the electron distribution which is visualized after tentatively assigning the unknown phases to the measured structure factors. However, crystallographers rarely push the structure modeling much further and do not investigate the finer details of electron density in between atoms and molecules, although this is nowadays much more easily available thanks to more powerful radiation sources (including synchrotron radiation) and more sensitive and faster detectors. Pushing this limit would enable extracting more information on the robustness of a given synthon, of a given metal-ligand coordination or a host-guest interaction, which are quite important in crystal engineering.

In parallel to the experimental side, also the *ab initio* quantum chemical analysis of these materials made enormous progresses in the past decade, which enable calculation of a number of properties that are not straightforwardly available from measured scattering intensities.

In the future, one may expect that the whole process of material design and fabrication could be integrated, having as initial step predictions based on calculated or measured electron density models of the molecular building blocks, that could offer an initial guess of the expected property of a crystalline solid.

### Endnotes

^a^It is always important to remember that the electron density is a probability function to find one electron in space, therefore it is always positive, despite the charge of the electron is actually negative. Thus an excess of electron density in an atom means a negative charge.

## Authors’ original submitted files for images

Below are the links to the authors’ original submitted files for images. Authors’ original file for figure 1 Authors’ original file for figure 2 Authors’ original file for figure 3 Authors’ original file for figure 4 Authors’ original file for figure 5 Authors’ original file for figure 6 Authors’ original file for figure 7 Authors’ original file for figure 8 Authors’ original file for figure 9 Authors’ original file for figure 10
