# Supplementary figures and images for: Charge density analysis for crystal engineering
Source: Chem Cent J. 2014 Dec 16;8:68. doi: 10.1186/s13065-014-0068-x (PMC4266768; doi:10.1186/s13065-014-0068-x)

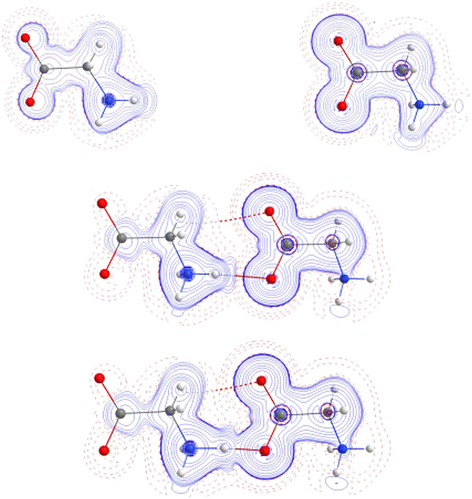

Supplement: Supplementary file 1 — Authors’ original file for figure 1 [file 13065_2014_68_MOESM1_ESM.gif]

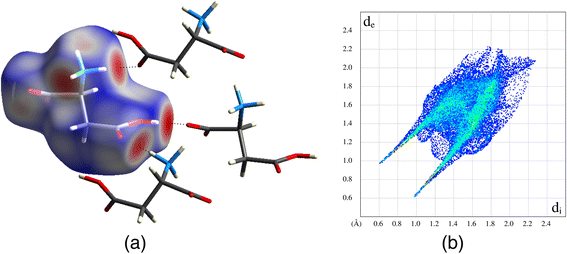

Supplement: Supplementary file 2 — Authors’ original file for figure 2 [file 13065_2014_68_MOESM2_ESM.gif]

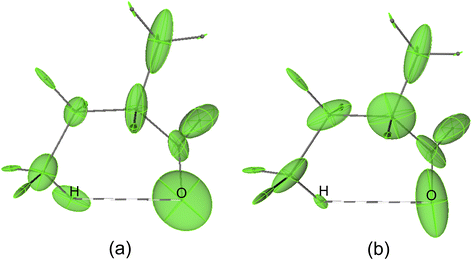

Supplement: Supplementary file 3 — Authors’ original file for figure 3 [file 13065_2014_68_MOESM3_ESM.gif]

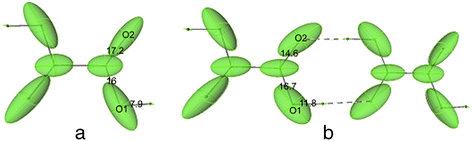

Supplement: Supplementary file 4 — Authors’ original file for figure 4 [file 13065_2014_68_MOESM4_ESM.gif]

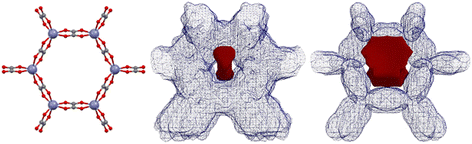

Supplement: Supplementary file 5 — Authors’ original file for figure 5 [file 13065_2014_68_MOESM5_ESM.gif]

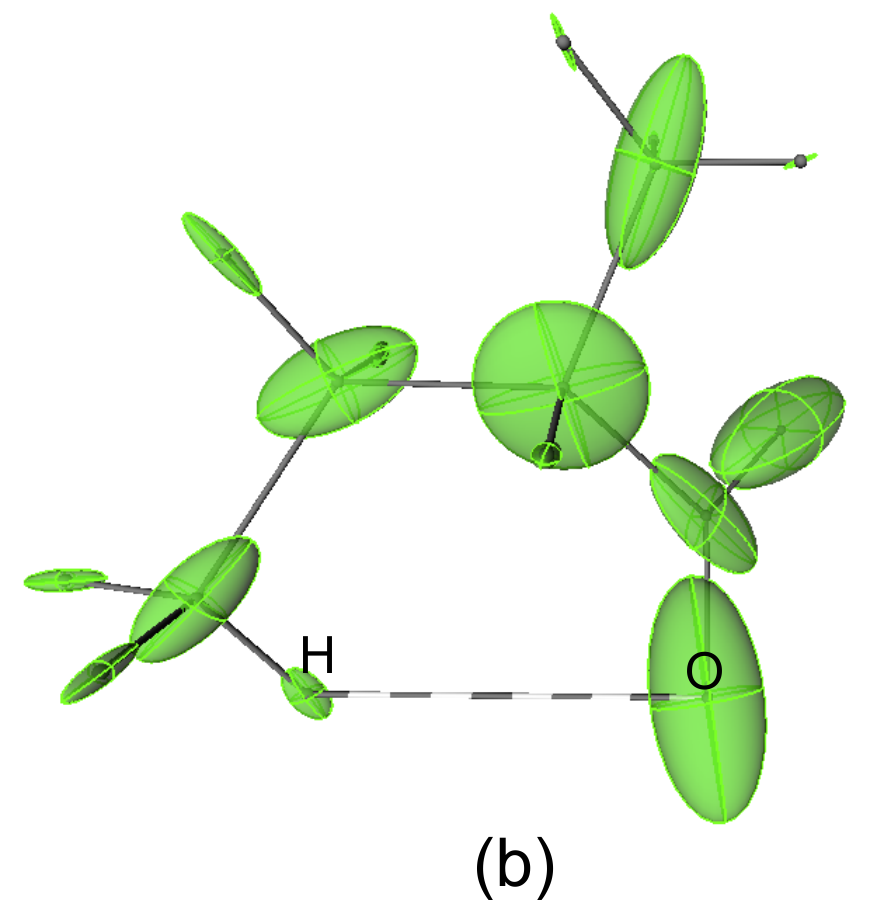

Supplement: Supplementary file 7 — Authors’ original file for figure 7 [file 13065_2014_68_MOESM7_ESM.tiff]

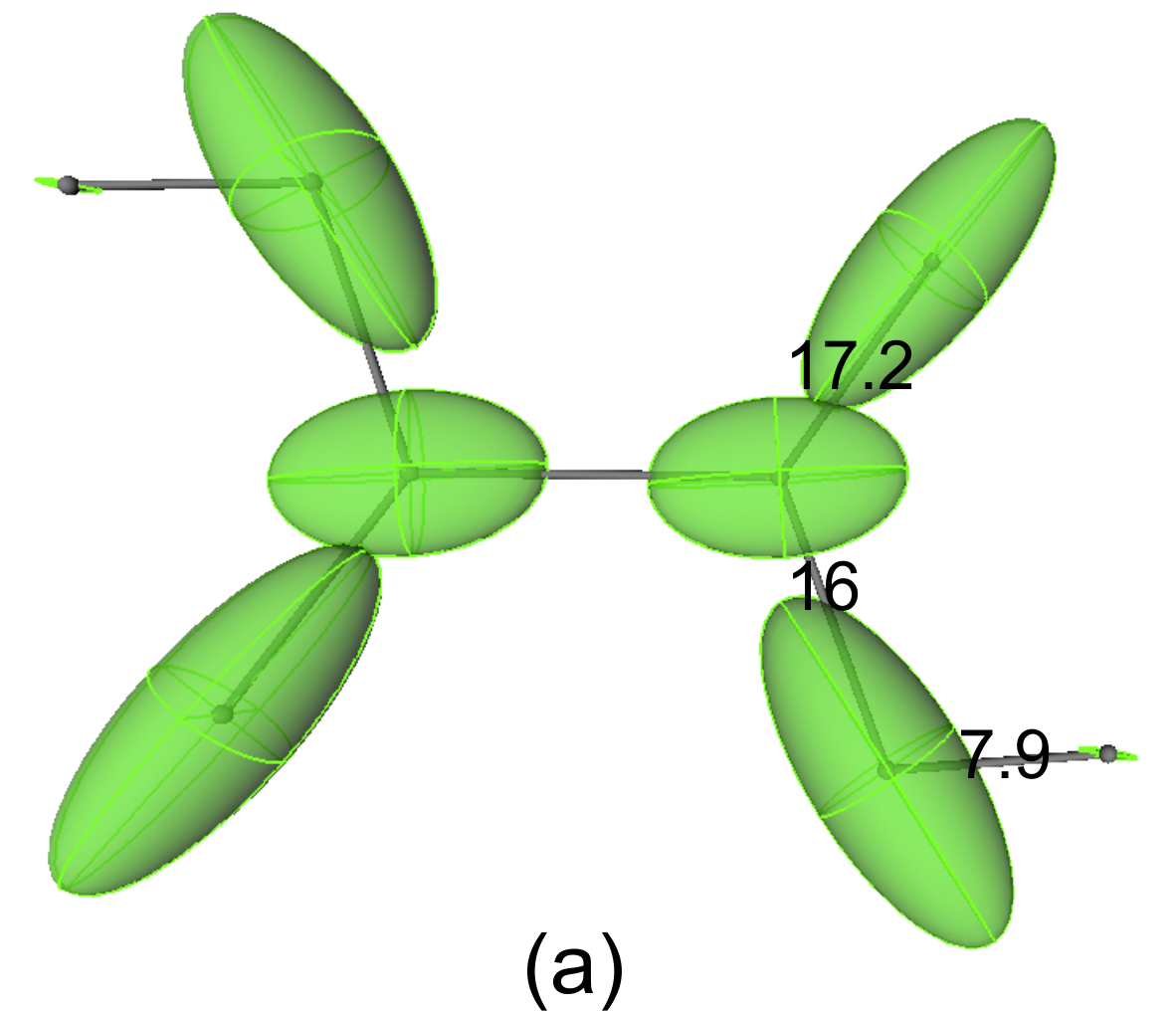

Supplement: Supplementary file 8 — Authors’ original file for figure 8 [file 13065_2014_68_MOESM8_ESM.tiff]

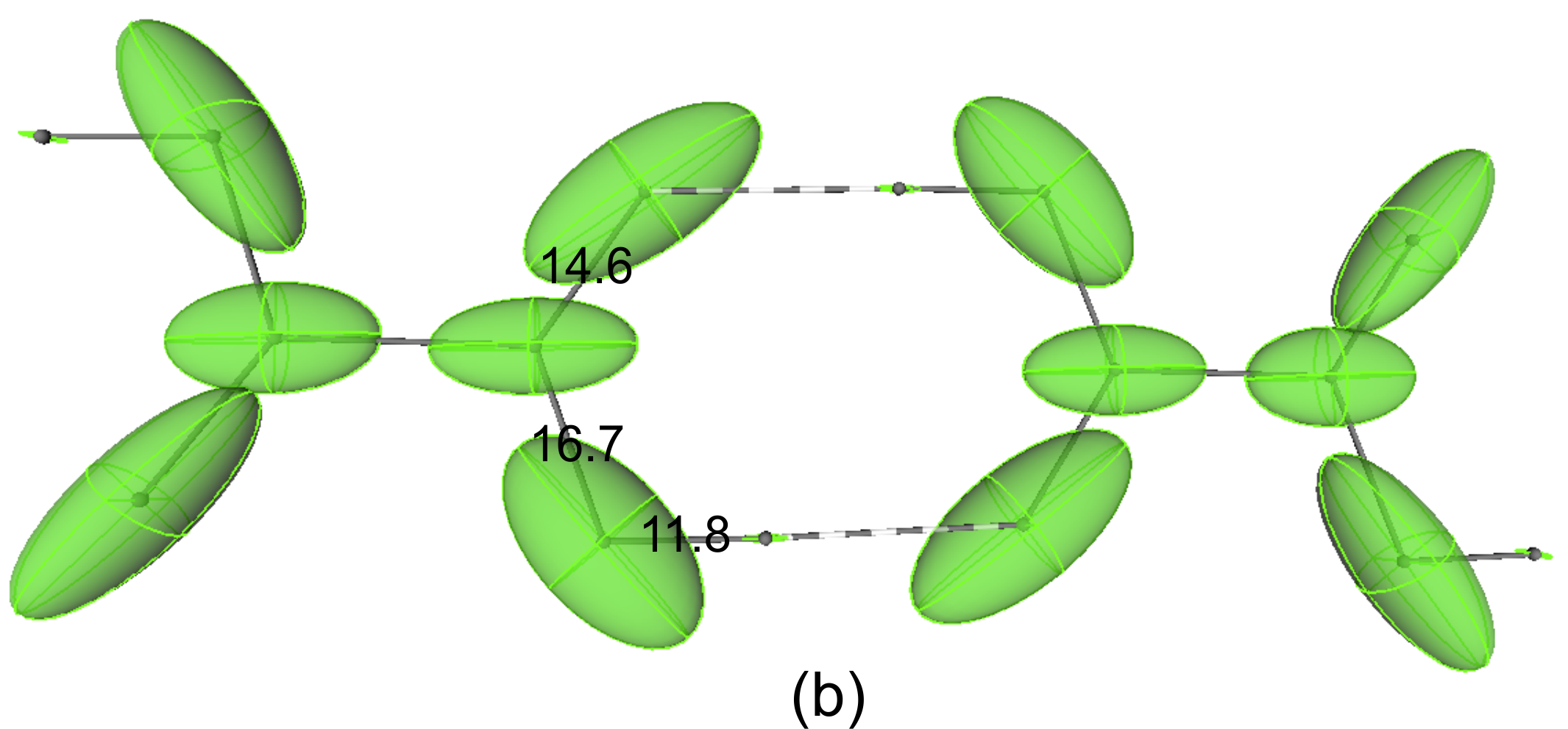

Supplement: Supplementary file 9 — Authors’ original file for figure 9 [file 13065_2014_68_MOESM9_ESM.tiff]

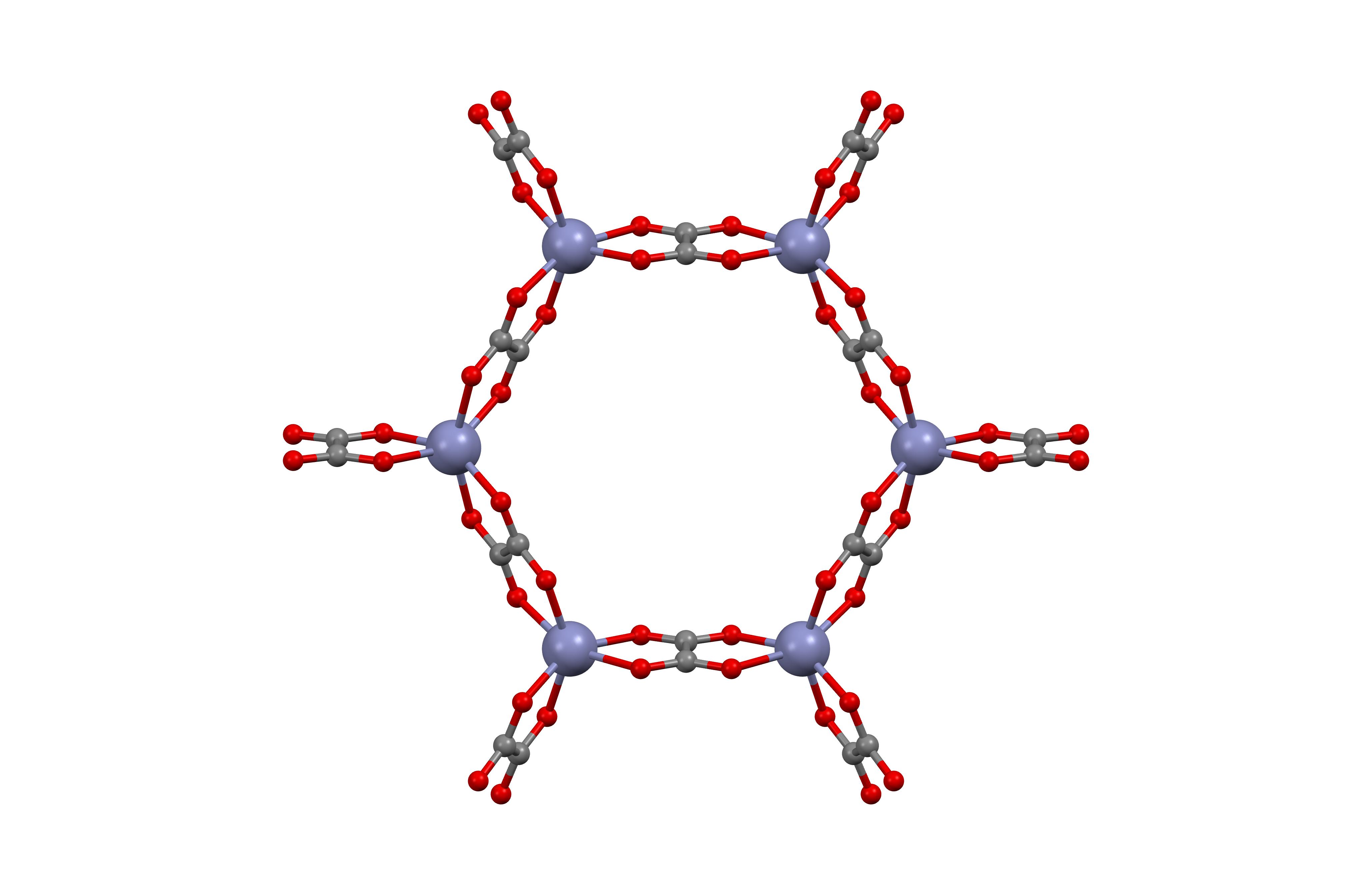

Supplement: Supplementary file 10 — Authors’ original file for figure 10 [file 13065_2014_68_MOESM10_ESM.jpeg]
